# Cryopreserved amniotic membrane and umbilical cord particulate matrix for partial rotator cuff tears: A case series

**DOI:** 10.1097/MD.0000000000016569

**Published:** 2019-07-26

**Authors:** J. Freeland Ackley, Michael Kolosky, Danielle Gurin, Robert Hampton, Richard Masin, David Krahe

**Affiliations:** a Twinsburg Family Health and Surgery Center, Twinsburg, OH; b Southwest Sports Medicine, Waco, TX.

**Keywords:** amniotic tissue, biologic factor, injury, joint, pain, rotator cuff, shoulder, tendon, umbilical cord

## Abstract

Amniotic membrane (AM) and umbilical cord (UC) are well known to have anti-inflammatory properties and have been shown to promote healing in various orthopedic indications. This study investigated whether intra-articular injection of AM/UC particulate matrix promotes healing of partial rotator cuff tears (RCTs).

A case series was performed on 10 patients that received injection of 50 mg AM/UC for partial RCTs that were refractory to conservative treatment. Outcomes included Penn Shoulder Score (PSS) questionnaire, range of motion examination, and magnetic resonance imaging (MRI) analysis before and at 6 months. Final MRI analysis was performed by a musculoskeletal radiologist in a blinded fashion.

Average PSS score (out of 100) increased from 46.8 ± 23.7 at baseline to 82.0 ± 19.1 at 6 months. The average PSS sub-scores of pain, satisfaction, and function increased 78.4%, 37.1%, and 82.3% from baseline, respectively. The subject's range of motion was 77.9% at baseline and increased to 99.9% at 6-months. Follow-up MRI scans did not demonstrate any significant change in RCT size. No adverse events were noted.

This small case series provides preliminary data for use of cryopreserved AM/UC particulate matrix in patients with refractory partial RCTs.

## 1 Introduction

The rotator cuff is a group of 4 muscles whose tendons attach to and form the covering around the head of the humerus. The primary function of the rotator cuff is to maintain glenohumeral joint stability and control its translation during shoulder movement. Hence if torn, the tendon's function would become disrupted and lead to shoulder instability, pain, reduced range of motion, and reduced functionality.^[1]^ The prevalence of rotator cuff tears (RCTs) is widespread however its incidence is known to increase with age, with up to 30% of the population older than 60 years having some form of an RCT.^[2–5]^

Once a RCT has occurred, its prognosis is hard to predict but is more likely to increase in size due to the continuous mechanical tension and lack of regenerative capability of the tendon.^[6–9]^ As the first standard of care for partial RCTs, nonsurgical management consisting of rest, activity modification, physical therapy, NSAIDs, and/or steroid injections is usually performed for 3 months to relieve pain and improve function before surgery is considered.^[10–12]^ However, it is recognized that prolonged nonsurgical management in symptomatic patients can have negative consequences including increase in RCT size, tear retraction, increased difficulty of repair and increased risk of muscular atrophy and fatty infiltration.^[6,13]^ This is also further complicated by the patient's continued pain, limitation in activities of daily living and overall dissatisfaction with the non-operative management; therefore, the choice of surgery largely remains up to the patient taking into account their symptoms, activity level, life goals, and medical co-morbidities.^[14]^ Bursal sided tears greater than 25% of the medial-lateral footprint, articular-sided tears greater than 50%, acute full-thickness ruptures, and failure of non-operative management historically are relative indications for operative intervention.^[8,15–17]^ Clinical improvement can be anticipated after surgical management with healing rates ranging from 80% to 95%, regardless the type of procedure such as debridement or repair performed arthroscopically or in open surgery.^[18–22]^ Despite these outcomes in RCTs, surgical management is controversial and presents inherent risks of infection, permanent stiffness of the joint, and a lengthy recovery time that ranges anywhere from 3 to 6 months post-operatively.^[18,23]^ Due to the aforementioned limitations of conventional therapy, alternative treatment methods are sought to improve the condition of patients suffering from partial RCTs without introducing the possibility of negative side effects. One well studied alternative is platelet-rich plasma (PRP) however, review of level I and II studies have found minimal clinical difference with its application.^[24–27]^ Alternatively, amniotic membrane (AM) and umbilical cord (UC) tissue have emerged as potential solutions. The unique anti-inflammatory and anti-scarring properties of this birth tissue have encouraged many to use it for multiple clinical applications in ophthalmology,^[28]^ non-healing skin ulcers and burns, and many surgical reconstructive procedures.^[29–31]^ AM/UC has also shown to promote healing in orthopedic indications,^[32,33]^ including when used as a tendon wrap to prevent inflammation and formation of adhesions.^[29,31,34,35]^ Within the matrix of cryopreserved AM and UC tissues, there exists a unique glycoprotein complex termed the HC-HA/PTX3 complex that has been found to be responsible for many of the anti-inflammatory and anti-scarring actions of these tissues.^[36]^ We thus speculated that AM/UC might aid in the healing of partial RCTs through such healing action. Unfortunately, there has been a limited number of human trials for this particular application despite the numerous laboratory and pre-clinical animal studies. Hence, in this pilot study, we evaluated the effects of intra-articular injections of cryopreserved human AM/UC particulate matrix in patients suffering from partial RCTs refractory to conventional medical treatments as a non-surgical therapy to promote healing, improve patient's quality of life, and prevent the need for surgical intervention.

## 2 Materials and methods

### 2.1 Patients

After approval by the Institutional Review Board (Cleveland Clinic IRB#16–125), patients were identified by retrospectively reviewing medical records at a local community hospital. Eligible patients had to have:

(1) a magnetic resonance imaging (MRI)-documented partial rotator cuff tear according to the treating physician and original MRI report,
(2) remained symptomatic despite non-surgical treatments including rest, physical therapy, NSAIDs, and/or steroid injection, and
(3) went on to receive intra-articular injection of cryopreserved AM/UC product (CLARIX FLO, Amniox Medical Inc., Atlanta, GA) from 1 of the general orthopedic surgeons/authors.

A total of 35 patients were then contacted by mail and telephone to see if they were interested in the study. A total of 11 patients were reached, a written informed consent was obtained, and the rights of subjects were protected. Of the 11 patients, 10 of them returned to the clinic for a 6 month follow up visit after AMUC injection for evaluation. The study was conducted in accordance with the Health Insurance Portability and Accountability Act (HIPAA) and Declaration of Helsinki.

### 2.2 Intra-articular injection and other treatments

All patients underwent the same treatment. In brief, under fluoroscopic visualization in the operating room, 50 mg of AM/UC particulate matrix was reconstituted in 1.5 cc of 0.5% Marcaine and then intra-articularly injected into the affected shoulder. Omnipaque radiocontrast (GE Healthcare, Chicago, IL) was used to ensure intra-articular AM/UC injection. After being placed in an arm immobilizer for 1 week following injection, the patient was allowed full arm range of motion (ROM) with no lifting, pushing or pulling greater than 1 lb. initially and then progressed through outpatient physical therapy utilizing phase II through IV rotator cuff repair rehabilitation protocol under the supervision of a local physical therapist.^[37]^

The cryopreserved AM/UC product used in this study has been commercially available since 2013 in the United States as a 361 human cell and tissue-based product. The cryopreserved AMUC particulate is derived from donated human placental tissue following healthy, live, caesarian section, full-term births which are then cleaned of blood under aseptic conditions. The AM and UC are dissected from the placenta proper, morselized, lyophilized, and terminal sterilized. The product comes as a dry powder in small vial stored at room temperature.

At baseline and 6-months post-AM/UC injection, patients filled out the Penn Shoulder Score (PSS) questionnaire, which is a comprehensive and validated 100-point scale capturing the patients’ pain, satisfaction, and abilities to perform daily tasks and sports-related activities.^[38,39]^ In addition, they also received an MRI and physical examination to assess the ROM using a goniometer in all basic ranges of the shoulder (e.g. abduction, flexion, internal, and external rotation). ROM was compared to healthy ROM as defined as 180° for forward flexion and abduction, 70° for internal rotation, and 90° for external rotation.^[40]^ This was performed by the same individual pre and post-injection. The MRI was performed with a 3 Tesla scanner (Siemens TIM Trio MRI scanner, Washington, DC) with coronal (proton density and T2) and sagittal (proton density and T2) views with a 16 cm field of view, 3 mm slice thickness and 256 × 512 matrix.

Aside from the treating physician's analysis and the initial radiologist interpretation, the MRI analysis was also performed by a musculoskeletal radiologist who had been blinded to any identifiable factors of the patient to validate the findings. The grading criteria for the masked review by the musculoskeletal radiologist included measuring the rotator cuff (supraspinatus) tendon tears (size, depth, signal intensity, and surface involvement) and the presence and absence of effusion, synovitis, and capsulitis. The RCT size was determined based on the medial–lateral (ML) tear dimension (in mm) and depth measured on T2 coronal image. The partial RCT depth was graded as ≤50% or >50%, signal intensity was graded as fluid intensity, increased PD/T2 but not fluid, or normal, and surface involvement was determined to be articular or bursal. The anteroposterior (AP) tear dimension (in mm) was measured on T2 sagittal. Lastly, the infraspinatus and subscapularis tendons were graded as normal or having tendinosis.

### 2.3 Statistical considerations

To assess the efficacy of this treatment, the patient's Penn Shoulder Score (PSS), ROM according to physical examination, and MRI analysis were compared before and 6 months after AM/UC injection. All data, including PSS scores, ROM evaluation, limitations seen at the follow-up visits, and functional status and remaining difficulties with Activities of Daily Living (ADLs) were stored in REDCap (Vanderbilt University). Finally, paired *t* test analysis was performed to compare the PSS scores, ROM, and subjective outcomes from the patient. Data were normally distributed and recorded as mean ± standard deviation. All analyses were performed using the R statistical programming language (R version 3.3.3 (2017–03-06), Vienna, Austria; R Core Team, 2015). This was a pilot study and sample size was determined based on published recommendation of 10-30 patients in the literature.^[41,42]^ A *P* value of less than .05 was considered statistically significant.

## 3 Results

### 3.1 Clinical features

A total of 10 patients (5 males, 5 females, average age 55.9 ± 11.8) were enrolled in the study. One patient was lost to follow-up and was excluded from the data analysis. Patients were symptomatic for 6.5 ± 4.2 months (range 1.5–12 months) prior to injection despite receiving non-surgical treatments including rest (10/10), physical therapy (8/10), NSAIDs (7/10), steroid injections (7/10), and opioids (1/10).

As shown in Table 1, the baseline PSS showed an average pain score of 13.4 ± 9.5 (out of 30), an average satisfaction score of 6.2 ± 4.5 (out of 10), and an average function score of 27.2 ± 13.9 (out of 60). Collectively, these values yielded an initial, average overall PSS score of 46.8 ± 23.7 (out of 100). The baseline ROM measurement showed an average of 85 ± 15%, 73 ± 23%, 90 ± 53%, and 64 ± 35% of healthy shoulder range of motion for forward flexion, abduction, internal rotation, and external rotation, respectively.

**Table 1:**
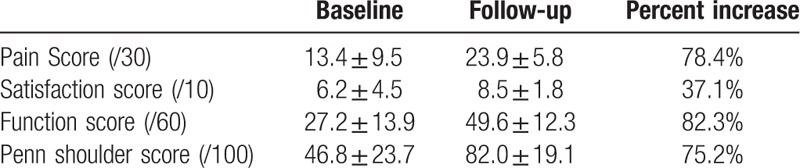
Penn shoulder scores.

Pre-injection MRI results confirmed partial RCTs as evidenced by damage to the supraspinatus. However, based on the blinded MRI interpretation, 1 patient had tendinosis of the supraspinatus while another patient's tendon appeared normal. Of those patients with confirmed RCTs by the treating physician and radiologist in our study, the average AP and ML tear dimensions were 10.0 ± 7.7 and 12.3 ± 6.7 mm, respectively. Five patients presented with partial tears >50% (3 articular, 2 bursal) and 3 patients presented with partial tears ≤50% (2 articular, 1 bursal). In addition, many of the patients presented with infraspinatus tendinosis (75%), subscapularis tendinosis (50%), moderate effusion (12.5%), synovitis (12.5%), and capsulitis (25%) of the joint.

### 3.2 Improvement after Injection of AM/UC

At the 6 month follow up visit, the PSS score (Table 1) showed an average pain score of 23.9 ± 5.8 which represented a 78.4% increase from the baseline, an average satisfaction score of 8.5 ± 1.8 which represented an increase of 37.1% from the baseline, and an average function score of 49.6 ± 12.3 which represented a 82.3% increase from the baseline. The combined PSS improved from 46.8 ± 23.7 at the baseline to 82.0 ± 19.1 at the 6-month follow up visit, which represented a 75.2% increase. The ROM at the 6-month follow up visit (Table 2) was at an average of 95 ± 14% (forward flexion), 91 ± 18% (abduction), 120 ± 18% (internal rotation), and 94 ± 7% (external rotation) of the full healthy shoulder range of motion. This represented a 28% increase in overall shoulder ROM when compared to the baseline value. In addition, the follow-up physical examination also revealed that patients noted diminished or absent pain in activities of daily living following injection. More specifically, none of these patients could perform their usual sport or hobby without difficulty before injection. However, after AM/UC injection, 70% of patients reported no difficulties at all in completing these activities, thus resulting in an increase of their quality of life. Recurring pain was typically a result of intensive activity or sleeping on the affected side. No adverse events or reactions were noted during the study period.

**Table 2:**
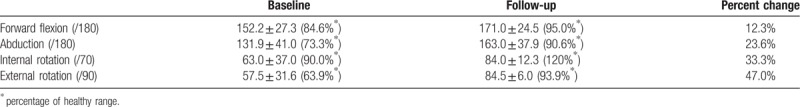
Range of motion data.

**Figure 1:**
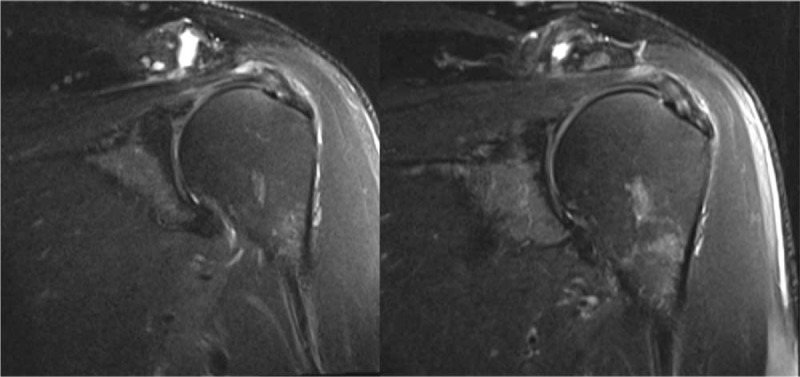
Representative MRI images of rotator cuff pre- and post-injection. Improved signal intensities in the supraspinatus and to a lesser extent the infraspinatus. MRI = magnetic resonance imaging.

The MRI results showed an insignificant change (*P* >.05) in rotator cuff size of 10.4 ± 7.4 mm (AP) and 13.4 ± 8.0 mm (ML). There was also no change in the depth of the rotator cuff tears, however, 2 of the 4 cases with fluid intensity at baseline decreased to PD/T2 at 6 months (Fig. 1). In addition, the 1 subject with moderate effusion at baseline decreased to small effusion and all cases showed absence of capsulitis at 6 months. There was no change in presence of synovitis nor subscapularis diagnosis, however, 1 additional case of infraspinatus tendinosis was diagnosed at 6 months.

## 4 Discussion

Rotator cuff tears are the leading causes of shoulder pain, accounting for more than 4.5 million physician visits annually.^[43]^ Corticosteroid injections remain 1 of the most commonly used treatments for chronic tendon disorders,^[12]^ however there are some controversies including

(1) a lack of clinical studies evaluating their use for partial RCTs,
(2) there is not a defined dosage regimen,
(3) they have been shown to promote full thickness tears within 12 weeks and
(4) are usually short-lasting.^[44,45]^

More recently, platelet-rich plasma and mesenchymal stem cells (MSC) have been investigated for their use in RCTs. Most studies have evaluated the use of PRP in conjunction with arthroscopic RCT repair but results are contrasting.^[22,46–48]^ One study has evaluated MSCs in full rotator cuff tear repair however it was also through surgical intervention in 14 patients.^[49]^ In regards to non-surgical treatment, PRP has been shown to have better effects than steroids at 12 weeks, although statistically significant results were not achieved at any time thereafter. ^[50–52]^ Herein, we surmise that non-surgical treatment with corticosteroid and PRP injections for partial RCTs and tendinosis have limited clinical benefit after 12 weeks from procedure. By indirect comparison, we note that intra-articular injection of AM/UC particulate matrix was effective in reducing pain and improving function in the shoulders of patients suffering partial RCTs that were refractory to conservative non-surgical treatments through the duration of the study at 6 months. This was demonstrated by a notable 75.2% increase in the average Penn Shoulder Score and a 28.1% increase in the average ROM.

Despite the symptomatic relief experienced by these refractory patients, follow-up MRI scans did not demonstrate any significant change in the RCT size as anticipated. This potentially could be due to the short follow up period of 6 months, limited sample size, or difficulty in MRI interpretation.^[53,54]^ Also, while we do not fully understand why 1 person had increased tendonosis, we hypothesize this may be attributed to the previously mentioned limitations of the MRI as their particular PSS and function improved. This present study showed an absence of any MRI evidence of tear progression, whereas numerous other studies have shown partial RCTs often likely to increase in size and progress to full-thickness tears.^[7,6–9]^ This coupled with the symptomatic relief suggests an overall clinical benefit.

The aforementioned clinical benefit may be due to the known anti-inflammatory and anti-scarring actions of AM/UC.^[25]^ During the tendon repair process, an initial inflammatory phase is followed by a proliferative and remodeling stage that is usually characterized by irregular inflammation, collagen disposition, and fibrovascular scar tissue with a large proportion of type III collagen that is eventually remodeled to type I collagen. It is during this proliferative and remodeling stage in which mesenchymal stem cells (MSCs) are recruited to the area to differentiate into tenocytes and myofibroblasts. In a large study by Hernigou et al, there was a significant decrease in MSCs present in the greater tuberosity of patients with rotator cuff disease and affected by a number of clinical factors.^[55,56]^ However, AM was shown to reduce the number of inflammatory cells during tendon repair which facilitated the organization and alignment of a regular collagen matrix. ^[35]^ More recently, the HC-HA/PTX3 complex has been identified in cryopreserved AM/UC and has been demonstrated to be responsible for several of the tissue's anti-inflammatory and anti-scarring therapeutic actions, including inducing apoptosis of pro-inflammatory cells, inhibiting the differentiation of myofibroblasts, and modulating the local inflammatory cytokine signaling milieu.^[36,57]^ Hence the AM/UC matrix may allow for resolution of chronic inflammation helping to promote wound healing.

If these results can be replicated on a larger scale, not only would the patient benefit from functional and pain improvement but there may also be a positive socioeconomic impact. This can be extrapolated from a study by Mather et al where they found societal cost savings approach $3.44 billion per year when patients undergo rotator cuff repairs compared to conservative therapies.^[58]^ While individual costs vary per institution, a published cost-analysis from 1 fellowship trained sports medicine orthopedic surgeon at an outpatient academic center found the mean charge to insurers was $31,459.35 with a mean reimbursement of $9679.08. This included the direct surgical, hospital overhead, and indirect costs of which $3432.67 was attributed to anchor implants alone.^[59]^ Using the AM/UC injection in place of arthroscopic rotator cuff repair would decrease operative time and implant costs thus significantly lowering the hospital overhead and direct surgical costs and adding to the $3.44 billion saved yearly. Although this was not addressed in this study, further potential savings could be applied if done as an office procedure with ultrasound guidance avoiding hospital and ambulatory surgical center costs altogether.

While this is a pilot study, there are several weaknesses that we would need to address. First, we would need to establish several control groups to allow for a randomized prospective study and reduce potential influence of confounding variables (co-morbidities, duration, RCT size, etc). Given the correlations we referenced earlier, all patients enrolled would be randomized into either placebo, corticosteroid, or AM/UC injection followed by a typical 6-month phase II through IV physical therapy protocol and tracked for 12 months. MRI or ultrasound would then be utilized to assess for quality of tendon healing to supplement patient-reported outcomes such as the Penn Shoulder Score. Extending the length of study would allow for a more conclusive determination of rotator cuff healing and analyze if the improvements seen are sustainable. Second, the quantity and location of injection would need to be adjusted. Currently, the AM/UC injectable is available in 25, 50, and 100 mg quantities. Anecdotally from manufacturer, these have been applied in small, medium, and large joints respectively. For future study, 100 mg would be utilized and the injection would be injected intra-articular or subacromial based on the tear location. While the location of injection was standardized to intra-articular, all 3 bursal sided tears had improvement on par with the average for the group. The functional outcome score subset within the PSS saw a significant improvement of 82% but was overall low and could be attributed to both location and quantity of the injection. By localizing the injection to the pathology, we may have seen greater benefit. Additionally, utilization of additional outcome tools such as Constant Score and ASES would have further verified our results, however, there is a lack of consistency in the literature on which tools to use.^[60]^ Lastly, background variables such as: age, gender, smoking history, diabetes, BMI, autoimmune disorders, chronic steroid use, previous corticosteroid injection, previous physical therapy (and duration), and prior shoulder surgery would need to be tracked.

## 5 Conclusion

This small case series provides preliminary data for use of cryopreserved AM/UC particulate matrix in patients with refractory partial RCTs. This data is based on a limited sample size and further prospective studies using a large sample size and a control group is warranted to confirm this therapeutic benefit.

## Author contributions

**Conceptualization:** Freeland Ackley, Robert Hampton.

**Data curation:** Freeland Ackley, Danielle Gurin, Robert Hampton.

**Formal analysis:** Freeland Ackley, Michael Kolosky, Danielle Gurin, Robert Hampton, Richard Masin, David Krahe.

**Investigation:** Freeland Ackley, Danielle Gurin, Richard Masin, David Krahe.

**Methodology:** Freeland Ackley.

**Project administration:** Freeland Ackley, Robert Hampton.

**Resources:** Freeland Ackley.

**Writing – original draft:** Freeland Ackley, Michael Kolosky.

**Writing – review & editing:** Freeland Ackley, Michael Kolosky, Danielle Gurin, Robert Hampton, Richard Masin, David Krahe.

Freeland Ackley orcid: 0000-0002-6307-9903.
